# Community acquired multi-drug resistant clinical isolates of *Escherichia coli* in a tertiary care center of Nepal

**DOI:** 10.1186/s13756-015-0059-2

**Published:** 2015-05-01

**Authors:** Shamshul Ansari, Hari Prasad Nepal, Rajendra Gautam, Sony Shrestha, Puja Neopane, Ganga Gurung, Moti Lal Chapagain

**Affiliations:** Department of Microbiology, Chitwan Medical College Teaching Hospital, Bharatpur, Chitwan Nepal; College of Nursing, Chitwan Medical College Teaching Hospital, Bharatpur, Chitwan Nepal

**Keywords:** AmpC, ESBL, *Escherichia coli*, MBL, MDR, XDR

## Abstract

**Background:**

Multi-drug resistance (MDR) in Gram-negative organisms is an alarming problem in the world. MDR and extensively-drug resistance (XDR) is in increasing trend due to the production of different types of beta (β)-lactamases. Thus the aim of this study was to document the incidence of MDR and XDR in clinical isolates of *Escherichia coli* and also to find out the enzymatic mechanisms of β-lactam antibiotics resistance.

**Methods:**

Two hundred clinical isolates of *Escherichia coli (E. coli)* identified by standard laboratory methods were studied. Antibiotic susceptibility profile was performed for all the isolates and the suspected isolates were phenotypically tested for the production of extended spectrum β-lactamase (ESBL), metallo β-lactamase (MBL) and AmpC β-lactamase (AmpC) by recommended methods.

**Results:**

Around three-fourth (78%) of the total isolates were multi-drug resistant. ESBL, MBL and AmpC production was found in 24%, 15% and 9% of isolates respectively. Amikacin, chloramphenicol and colistin were found to be the most effective antibiotics.

**Conclusions:**

High percentage of MDR was observed. β-lactamase mediated resistance was also high. Thus, regular surveillance of drug resistance due to β-lactamases production and infection control policy are of utmost importance to minimize the spread of resistant strains.

## Background

Resistance to broad spectrum β-lactams, mediated by extended spectrum β-lactamases (ESBL), metallo β-lactamases (MBL) and AmpC β-lactamases (AmpC) enzymes is an increasing problem worldwide [1]. Presence of the latter two enzymes in clinical infections can result in treatment failure if one of the second- or third-generation cephalosporin is used. The scenario worsens in cases of MBL production where the drugs of last resort the carbapenems are rendered inactive [2]. Due to extensive use of β-lactam antibiotics over the last several decades in the clinical practice, various β-lactamases have emerged. ESBLs are the enzymes produced by Gram-negative bacilli that have the ability to hydrolyze β-lactam antibiotics containing an oxyimino group (third generation cephalosporins and aztreonam) and are inhibited by β-lactamase inhibitors such as clavulanic acid, sulbactam and tazobactam [3].

ESBLs were first identified in 1983. Since the time, they have been found worldwide in a number of organisms, including *Klebsiella pneumoniae (K. pneumoniae), Klebsiella oxytoca, Escherichia coli (E. coli), Proteus mirabilis, Enterobacter cloacae, Morganella morganii*, *Serratia marcescens*, *Shigella dysenteriae*, *Pseudomonas aeruginosa*, *Burkholderia cepacia*, *Capnocytophaga ochracea*, *Citrobacter* species, and *Salmonella* species [4-10].

The emergence of ESBL producing bacteria, particularly *E. coli* and *K. pneumoniae*, is now a critical concern for the development of therapies against bacterial infection. The major ESBL producer was *K. pneumoniae* before 2000, but now *E. coli* has become an important ESBL carrier in Western countries [11-13]. Since the ESBL genes are usually found in large plasmids, they also contain other antimicrobial resistant genes. Therefore, most ESBL producing organisms are also resistant to aminogylcosides, fluororquinolones, tetracyclines, chloramphenicol, sulfonamides. Carbapenems are the mainstay of therapy for infections caused by ESBL producing organisms [14]. Therefore, resistance against these agents poses therapeutic challenge. Based on molecular studies, carbapenem hydrolyzing enzymes are classified into four groups A, B, C and D. The MBLs belong to group B and are enzymes requiring divalent cations as cofactors for enzyme activity, being inhibited by the action of a metal ion chelator [15]. The MBLs efficiently hydrolyze all β-lactams, except aztreonam in vitro [16].

AmpC-mediated β-lactam resistance in *E. coli* and *Klebsiella* spp. is an emerging problem [17]. High level AmpC production is typically associated with in vitro resistance to all β-lactam antibiotics except for carbapenems and cefepime. In addition, treatment failures with broad-spectrum cephalosporins have been documented [18,19].

The production of β-lactamases is the single most prevalent mechanism responsible for resistance to β-lactams among clinical isolates belonging to the family of Entrobacteriaceae [20]. Over the years, many new β-lactam antibiotics have been developed. However, with each new class of antibiotics, a new β-lactamase has emerged and caused resistance to it. Presumably, the selective pressure imposed by the use and overuse of new antibiotics in the treatment of patients has resulted in the emergence of new variants of β-lactamases [21]. In this perspective, the present study was designed to document the existence of ESBL, MBL and AmpC-production in clinical isolates of multi-drug and extensively-drug resistant *E. coli* and to determine whether any alternative regimens are available to treat them.

## Methods

A total of 200 random, non-redundant and non-repetitive community acquired clinical isolates of *E. coli* (urine 90, sputum 50, pus 40, blood 20), identified by standard microbiological technique [22] in the bacteriological laboratory of Chitwan Medical College Teaching Hospital (CMCTH), a 600 bed tertiary care center of central Nepal in 2013, were subjected to phenotypic determination of the antimicrobial susceptibility, identification of MDR, XDR and pan drug resistant (PDR); and detection of ESBL, MBL and AmpC β-lactamase production.

### Antimicrobial susceptibility testing

Antibiograms of the isolates were determined by modified Kirby-Bauer disk diffusion method on Mueller-Hinton agar standard media using commercially prepared disks (HiMedia Laboratories Pvt. Limited, India) in compliance with Clinical and Laboratory Standards Institute (CLSI) guidelines [23]. Antimicrobials used were: penicillin [ampicillin (10 μg)], penicillins with β-lactamase inhibitors [ampicillin-sulbactam (10/10 μg), amoxicillin-clavulanic acid (10 μg)], narrow spectrum cephalosporin [cefazolin (30 μg)], extended spectrum cephalosporins [ceftazidime (30 μg), ceftriaxone (30 μg), cefepime (30 μg)], cephamycin [cefoxitin (30 μg)], antipseudomonal penicillins with β-lactamase inhibitors [piperacillin-tazobactam (100/10 μg), ticarcillin-clavulanic acid (75/10 μg)], monobactam [aztreonam (30 μg)], carbapenems [imipenem (10 μg), meropenem (10 μg)], aminoglycosides [gentamicin (10 μg), amikacin (30 μg), tobramycin (10 μg)], fluoroquinolones [ciprofloxacin (5 μg), ofloxacin (5 μg)], folate pathway inhibitor [co-trimoxazole (25 μg)], phenicol [chloramphenicol (30 μg)] and polymyxin [colistin (10 μg)]. Interpretation of susceptibility was made according to the tables for interpretative zone diameters of CLSI [23]. *E. coli* 25922 was used as a control organism for antibiotic sensitivity testing.

### Identification of MDR, XDR and PDR isolates

MDR, XDR and PDR isolates were identified according to the guidelines recommended by joint initiative of the European Centre for Disease Prevention and Control (ECDC) and the Centers for Disease Control and Prevention (CDC) [24]. According to the guidelines, the isolates showing non-susceptibility to at least one agent in three or more antimicrobial categories were identified as MDR, non-susceptibility to at least one agent in all but two or fewer antimicrobial categories (i.e. bacterial isolates remained susceptible to only one or two categories) were identified as XDR and non-susceptibility to all agents in all antimicrobial categories were identified as PDR. To ensure correct application of these definitions, bacterial isolates were tested against all or nearly all of the commercially available antimicrobial agents within the antimicrobial categories (recommended by ECDC and CDC) and selective reporting and suppression of results were avoided [24].

### Phenotypic detection of ESBL

Isolates of *E. coli* were examined for their susceptibility to 3^rd^ generation cephalosporins by using ceftazidime (30 μg) and cefotaxime (30 μg) disks. The isolates showing diameter of ≤ 22 mm zone of inhibition for ceftazidime and/or ≤ 27 mm for cefotaxime were considered as ESBLs suspects as per National Committee for Clinical Laboratory Standards (NCCLS) guidelines [25]. All suspected isolates for ESBLs production were confirmed by the combination disk method on Mueller Hinton agar plates that were inoculated with standardized inoculums (comparable to 0.5 McFarland standards) of the isolates to form a lawn culture. Separate commercial disks containing cefotaxime (30 μg) and ceftazidime (30 μg) with and without clavulanic acid (10 μg) were placed over the lawn culture. An increase in zone size of more than or equal to 5 mm for cefotaxime and ceftazidime with and without clavulanic acid indicated ESBL production as described by Carter *et al*. [26].

### Phenotypic detection of MBL

Isolates that were found resistant to imipenem, meropenem or third generation cephalosporins (ceftazidime) in Kirby Bauer disk diffusion method were presumptively considered MBL producers and were confirmed by the imipenem disk with EDTA methods. Briefly, the test inoculums (comparable to 0.5 McFarland standards) were prepared and transferred on to Mueller Hinton agar plates. Two imipenem (10ug) disks were placed on the surface of agar plate and 10 μl EDTA solutions was added to one of them to obtain a desired concentration of 750 μg. Plates were incubated for 16 to 18 hours at 35°C. An increase in zone size of more than or equal to 7 mm for imipenem-EDTA disk compared to imipenem disk alone indicated MBL producer strain as described by Yong et al. [27].

### Phenotypic detection of AmpC

All *E. coli* isolates resistant to cefoxitin in Kirby-Bauer disk diffusion method were confirmed for AmpC β-lactamase production by modified Hodge test. In the test, a cefoxitin susceptible *E. coli* indicator strain (ATCC 25922) was plated on Muller Hinton agar medium and the cefoxitin disk was placed. Test organism was streaked toward the cefoxitin disk. If the test organism expressed AmpC, it hydrolyzed the cefoxitin and showed growth along the intersection of the streak and the zone of inhibition from the cefoxitin disk [28].

### Ethical consideration

The samples used in this study were from routine clinical specimens; however, verbal consent was taken from the patients. The ethical approval was obtained from Institutional Review Committee of Chitwan Medical College Teaching Hospital (CMCTH), Bharatpur, Nepal to conduct the study.

## Results

### Resistance pattern of *E. coli*

All of the *E. coli* isolates tested exhibited resistance to amoxicillin-clavulanic acid and 77 % of them remained resistant to ciprofloxacin whereas all the isolates were susceptible to colistin and few isolates (7%) were resistant to imipenem (Table 1).

**Table 1:** **Resistance rates of E. coli**

**Antibiotics**	**No. of resistant isolates (%)**	**MDR (n = 156)**	**XDR (n = 14)**
		**No. of resistant isolates (%)**	**No. of resistant isolates (%)**
**Penicillins**			
*Ampicillin*	148 (74)	130 (83.3)	14 (100.0)
**Penicillins with β-lactamase inhibitors**			
*Ampicillin-sulbactam*	114 (57)	100 (64.1)	14 (100.0)
**Narrow spectrum cephalosporins**			
*Cefazolin*	142 (71)	122 (78.2)	14 (100.0)
**Extended spectrum cephalosporins**			
*Ceftazidime*	104 (52)	84 (53.8)	14 (100.0)
*Ceftriaxone*	82 (41)	68 (43.6)	14 (100.0)
*Cefepime*	62 (31)	68 (43.6)	14 (100.0)
**Cephamycins**			
*Cefoxitin*	82 (41)	66 (42.3)	14 (100.0)
**Antipseudomonal penicillins with β-lactamase inhibitors**			
*Piperacillin-tazobactam*	48 (24)	34 (21.8)	14 (100.0)
*Ticarcillin-clavulanic acid*	114 (57)	100 (64.1)	14 (100.0)
*Amoxicillin-clavulanic acid*	200 (100)	156 (100.0)	14 (100.0)
**Monobactams**			
*Aztreonam*	72 (36)	58 (37.2)	14 (100.0)
**Carbapenems**			
*Imipenem*	14 (7)	0 (0)	14 (100.0)
*Meropenem*	74 (37)	60 (38.5)	14 (100.0)
**Aminoglycosides**			
*Gentamicin*	40 (20)	26 (16.7)	14 (100.0)
*Amikacin*	20 (10)	6 (3.8)	14 (100.0)
*Tobramycin*	44 (22)	32 (20.5)	12 (85.7)
**Fluoroquinolones**			
*Ciprofloxacin*	154 (77)	142 (91.0)	14 (100.0)
*Ofloxacin*	94 (47)	78 (25.0)	14 (100.0)
**Folate pathway inhibitors**			
*Cotrimoxazole*	118 (59)	102 (65.4)	14 (100.0)
**Phenicols**			
*Chloramphenicol*	34 (17)	20 (12.8)	12 (85.7%)
**Polymyxins**			
*Colistin*	0 (0)	0 (0)	0 (0)

### MDR and XDR isolate

Of total 200 *E. coli* isolates tested, 156 (78%) isolates were MDR and 14 (7%) isolates were XDR whereas no PDR isolate was identified (Table 1).

All MDR isolates were resistant to amoxicillin-clavulanic acid and 91% isolates were resistant to ciprofloxacin whereas amikacin, imipenem and colistin were found as the most effective antibiotics for the MDR isolates. All XDR isolates were resistant to most of the antibiotics tested whereas colistin was found as the effective regimen against all XDR isolates (Table 1).

### ESBL, MBL and AmpC producing isolates and their resistance profile

Among total tested isolates, the synthesis of ESBL, MBL and AmpC was detected in 48 (24%), 30 (15%) and 18 (9%) isolates respectively (Tables 2 and 3). Most of the antibiotics tested were non-effective against ESBL, MBL and AmpC producers whereas imipenem, amikacin, chloramphenicol and colistin were found effective regimens against ESBL producers and only colistin was effective against MBL and Amp C producing isolates (Table 2).

**Table 2:** **Resistance pattern of ESBL, MBL and AmpC producing isolates**

**Antibiotics**	**ESBL (n = 48)**	**MBL (n = 30)**	**Amp C (n = 18)**
	**No. of resistant isolates (%)**	**No. of resistant isolates (%)**	**No. of resistant isolates (%)**
Ampicillin	44 (91.7)	24 (80.0)	18 (100)
Ampicillin-sulbactam	44 (91.7)	24 (80.0)	18 (100)
Cefazolin	48 (100)	28 (93.3)	16 (88.9)
Ceftazidime	48 (100)	30 (100)	18 (100)
Ceftriaxone	48 (100)	24 (80.0)	12 (66.7)
Cefepime	36 (75.0)	16 (53.3)	0 (0)
Cefoxitin	34 (70.8)	26 (86.7)	18 (100)
Piperacillin-tazobactam	22 (45.8)	18 (60.0)	12 (66.7)
Ticarcillin-clavulanic acid	44 (91.7)	22 (73.3)	16 (88.9)
Amoxicillin-clavulanic acid	48 (100)	30 (80.0)	18 (100)
Aztreonam	48 (100)	22 (73.3)	12 (66.7)
Imipenem	2 (4.2)	14 (46.7)	4 (22.2)
Meropenem	32 (66.7)	22 (73.3)	12 (66.7)
Gentamicin	14 (29.2)	18 (60.0)	6 (33.3)
Amikacin	2 (4.2)	16 (53.3)	4 (22.2)
Tobramicin	16 (33.3)	16 (53.3)	6 (33.3)
Ciprofloxacin	46 (95.8)	28 (93.3)	18 (100)
Ofloxacin	40 (83.3)	24 (80.0)	14 (77.8)
Cotrimoxazole	40 (83.3)	24 (80.0)	12 (66.7)
Chloramphenicol	6 (12.5)	14 (46.7)	4 (22.2)
Colistin	0 (0)	0 (0)	0 (0)

**Table 3:** **Type of β-lactamases production among** ***E.coli*** **isolates**

**Type of β-lactamases**	**No. of producing isolates (%)**
ESBL	48 (24)
MBL	30 (15)
Amp C	18 (9)
ESBL + MBL	10 (5)
ESBL + Amp C	8 (4)
MBL + Amp C	6 (3)
ESBL + MBL + Amp C	4 (2)

### Multi-type β-lactamase production

Of the tested isolates, 10 (5%) were producers of both ESBL and MBL, 8 (4%) isolates synthesized both ESBL and AmpC whereas 6 (3%) isolates produced both MBL and Amp C. All the three types of β-lactamases (i.e. ESBL, MBL and AmpC) were detected in 4 (2%) isolates (Table 3).

### Resistance rates of antibiotics with different mode of action

All of the tested isolates were resistant to at least one cell wall inhibiting agents followed by folic acid metabolism inhibiting agent (59%) whereas no isolates were resistant to cytoplasmic membrane damaging agent (Table 4).

**Table 4:** **Resistance profile of isolates tested against 6 major classes of antibiotics with different mode of action**

**Mode of action**	**Antibiotic classes**	**No. of isolates resistant to at least one agent of antibiotic classes (%)**
**Inhibition of cell wall synthesis**	Beta-lactams	200 (100)
**Inhibition of protein synthesis**	Aminoglycosides	56 (28)
**Inhibition of DNA replication**	Fluoroquinolones	82 (41)
	Chloramphenicol	34 (17)
**Inhibition of folic acid metabolism**	Cotrimoxazole	118(59)
**Damaging of cytoplasmic membrane**	Polymyxins (colistin)	0 (0)

### Incidence of resistant isolates

Very minor numbers of isolates (4%) were resistant to only one antibiotic whereas majorities (85%) of isolates were resistant to at least three antibiotics (Table 5).

**Table 5:** **Incidence of isolates resistant to antibiotics**

**Resistant profile**	**No. of isolates (%)**
Resistant to only one antibiotic	8 (4)
Resistant to 2 antibiotics	22 (11)
Resistant to 3–20 antibiotics	170 (85)
**Total**	**200 (100)**

## Discussions

The production of $\beta$-lactamases as a major problem have drawn attention to a need for better diagnostic techniques and newer drugs to allow more specific therapy because of the emergence and spread of antimicrobial resistance. Therefore, the detection, characterization and antibiotic susceptibility pattern of $\beta$-lactamase producing organisms can lead to successful infection control, involving antimicrobial stewardship and public health interventions aimed at controlling the emergence of such life-threatening MDR bacteria. The global rise of MDR organisms and their production of resistant enzymes is a serious public health threat now a day.

There are various documented literatures on investigation of ESBL, MBL and AmpC producing isolates from Nepal. To the best of our knowledge, this is the first report from Nepal with an exclusive focus on investigating the current incidence and antimicrobial susceptibility patterns to a large number of antibiotics tested among ESBL, MBL and AmpC producing MDR and XDR *E. coli* isolates.

In current study we investigated 200 clinical isolates of *E. coli*, all of which were resistant to amoxicillin-clavulanic acid and 77 % of the isolates were found resistant to ciprofloxacin. Our finding is similar to the observation of Khanal et al. who reported that 66.7% of isolates were resistant to ciprofloxacin [29]. However, Khadgi et al. reported 100% resistance to ciprofloxacin [30]. Among the antimicrobials tested, colistin (100%), imipenem (93%) and amikacin (90%) were found as the most effective agents against *E. coli* in our study. Polymyxin (colistin) and amikacin as the most effective antibiotics has also been defined by other authors and it can be concluded that amikacin is a promising regimen for the empirical therapy [29-31]. Chloramphenicol is still an effective drug after so many years in use as only small percentage of isolates (17%) were resistant to it, a drug approved in 1947 for human clinical use.

In present study, of total isolates tested, 156 (78%) isolates were MDR. Similarly, higher rate of MDR was also reported by Sharma et al. (90.8%) [32] whereas Khanal et al. (50%) [29] and Baral et al. (38.2%) [31] reported its lower rate from Nepal. A high incidence of MDR *E. coli* was also observed by Ibrahim et al. (92.2%) from Sudan [33]. As it was a big concern for us, 7% of isolates exhibited XDR in the present study. However, we did not find any previous literature reporting XDR *E. coli* in Nepal.

All MDR isolates in this study were resistant to amoxicillin-clavulanic acid. Resistance to fluoroquinolones varies geographically and is an emerging problem in both developed and developing countries [34,35]. In the present study, MDR *E. coli* isolates showed relatively high resistance rates to ciprofloxacin (91%). This has been hypothesized to be related to the inappropriate use (over-use and miss-use) of fluoroquinolones for humans [36]. Also, prolonged use of low dose of the more potent fluoroquinolones such as ciprofloxacin has been shown to be the most significant risk factor for acquisition of resistance [37]. Majority of the MDR isolates were resistant to ampicillin (78%) and cotrimoxazole (65.4%). A few isolates also showed resistance to other antibiotics such as amikacin (3.8) and chloramphenicol (12.8) and no isolates were found to be resistant to imipenem and colistin. Likewise, Baral et al. from Nepal also reported similar resistance rates of ciprofloxacin (92.6%), ampicillin (94.1%), cotrimoxazole (86.8%), amikacin (6.2%) and chloramphenicol (27.1%) [31]. All XDR isolates were resistant to most of the antimicrobials tested whereas colistin was found as the most effective agent against all the XDR isolates.

ESBL-producing *E. coli* have been described in hospitals to cause various outbreaks, but their presence in community has also been documented [38-40]. The reduced susceptibility of Gram-negative isolates to the later generation cephalosporins could be attributable to ESBL or AmpC β-lactamase production or some other relevant underlying mechanisms. In this study, 48 (24%) ESBL and 18 (9%) AmpC β-lactamase producing *E. coli* strains were detected. Higher prevalence of ESBL producing *E. coli* has also been reported by other authors [31,41,42] but a lower prevalence of ESBL (8.6%) and similar prevalence of AmpC (7.8%) has been reported by Khanal et al. in Nepal [29]. This lower prevalence of ESBL by Khanal et al. may be due to the selection of only tracheal isolates from ICU. A higher prevalence of ESBL (67%) and AmpC (33%) has been reported by Altun et al. from Turkey [43]. This higher prevalence of ESBL and AmpC by Altun et al. may be because of the selection of various types of clinical samples from the ICU patients. MBL production is also in increasing trend causing the carbapenems to be the ineffective regimens. In our study we found, 30 (15%) MBL producers among tested isolates. Our finding is similar to 13.4% documented by Wadekar et al. from India [42].

Susceptibility pattern of ESBL-producing isolates showed that these strains are not only resistant to β-lactams but also to other classes of antibacterials including gentamicin, cotrimoxazole and fluoroquinolones. In this study, we found that the β-lactamases producing isolates were resistant to most of the antibiotics tested but amikacin, imipenem, chloramphenicol and colistin showed promising efficacy against ESBL and AmpC producing isolates, which is in accordance with the other report from Nepal [41]. The carbapenem group of antibiotics is the most effective regimen for ESBL and AmpC producers but the increasing rate of MBL production makes the serious problem in infection treatment. We observed that more than half of the MBL producers were resistant to nearly all the antibiotics tested while colistin was found effective against all the MBL producing isolates.

Multi type β-lactamases producing isolates were also reported in our study. Among total tested isolates, 10 (5%) isolates were producers of both ESBL and MBL. ESBL together with AmpC production was seen in 8 (4%) isolates, whereas, 6 (3%) isolates produced both MBL and AmpC and 4 (2%) isolates did all, ESBL, MBL and AmpC. Similar result of ESBL with AmpC production (5.4%) was also documented by Kaur et al. from India [44].

Over the past 20 years there has been increased resistance to β-lactam antibiotics due to production of ESBL mediated by plasmids. This type of resistance is now observed in all species of *Enterobacteriaceae* and currently disseminated worldwide [45]. In this study, no isolates were documented resistant to cytoplasmic membrane damaging agents (colistin) whereas all isolates were found resistant to at least one cell wall inhibiting agents (β-lactams) and provided a significant contribution for MDR. In addition, isolates resistant to protein synthesis inhibiting agents and DNA replication inhibiting agents seem to have contributed considerably in the production of MDR. Similar contribution of different antimicrobial agents was also noticed by Aly et al. in Egypt [46].

In the present study, we found that all the isolates were resistant to at least one agent and 170 (85%) isolates were resistant to at least 3 agents while 22 (11%) isolates were resistant to two agents and 8 (4%) isolates were resistant to only one antimicrobial agent.

## Conclusions

This study concluded that, there is high prevalence of community acquired MDR *E. coli* and high rate of ESBL, MBL and AmpC β-lactamase production in our set up. A strict hospital infection control policies and a prudent use of antimicrobial regimens are to be adopted by the concerned people to minimize the development of resistant strains. It is also essential to have a regular and routine monitoring of ESBL, MBL and AmpC β-lactamase producing clinical isolates in clinical laboratories. Regular nation wise surveillance of multidrug resistance seems necessary step to combat the severity of infections caused by MDR pathogens. Further studies at molecular level may be beneficial to rule out the cause of MDR pattern.
